# Syndemic Classes of Unhealthy Alcohol Use Reveal Inequities in Alcohol‐Related Consequences and HIV Care Outcomes

**DOI:** 10.1111/acer.70398

**Published:** 2026-09-17

**Authors:** Natalia Van Doren, Michael J. Silverberg, Varada Sarovar, Tory Levine, Alexandra Lea, Stacy A. Sterling, Vanessa A. Palzes, Felicia W. Chi, Andrea H. Kline‐Simon, Maha N. Mian, Elena Cyrus, Lunthita M. Duthely, Typhanye Dyer, Angela M. Heads, Michael Horberg, Janice Y. Tsoh, Derek D. Satre

**Affiliations:** ^1^ Department of Psychiatry and Behavioral Sciences University of California, San Francisco San Francisco California USA; ^2^ Division of Research Kaiser Permanente Northern California Pleasanton California USA; ^3^ Department of Psychology Suffolk University Boston Massachusetts USA; ^4^ Department of Population Health Sciences, College of Medicine University of Central Florida Orlando Florida USA; ^5^ Department of Obstetrics, Gynecology and Reproductive Sciences, and the Department of Public Health Sciences University of Miami School of Medicine Miami Florida USA; ^6^ Department of Epidemiology and Biostatistics, School of Public Health University of Maryland College Park Maryland USA; ^7^ Faillace Department of Psychiatry and Behavioral Sciences, McGovern Medical School University of Texas Health Science Center at Houston Houston Texas USA; ^8^ Mid‐Atlantic Permanente Research Institute Kaiser Permanente Mid‐Atlantic States Washington DC USA

**Keywords:** comorbidity, depression, health equity, HIV treatment, latent class analysis, smoking, unhealthy alcohol use

## Abstract

**Background:**

Unhealthy alcohol use among people with HIV (PWH) often co‐occurs with medical and mental health conditions and may negatively impact patient care. We examined syndemic classes of unhealthy alcohol use, substance use and comorbidities and their links to sociodemographic factors, alcohol treatment access, and HIV care outcomes.

**Methods:**

Participants were 4574 adult PWH in Kaiser Permanente Northern California who reported alcohol use on routine primary care screening between 2013 and 2020 (index date). Latent class analysis identified subgroups based on alcohol use level and co‐occurring conditions in the prior year, including smoking, alcohol and other substance use disorders, mental health diagnoses, chronic medical conditions, obesity, insufficient physical activity, and sexually transmitted infections. Age, race/ethnicity, sex, and HIV risk status were examined as predictors of class membership using latent class analysis (LCA) with covariates. HIV outcomes (viral suppression < 200 copies/mL, retention in care) and alcohol treatment in the year post‐index (psychotherapy, medication) were compared across classes using Bolck, Croon, and Hagenaars (BCH) three‐step procedure.

**Results:**

Four classes were identified: Heavy Use/Complex Behavioral Multimorbidity, Moderate Use/High Cardiometabolic Burden, Low Use/High Medical Morbidity, and Moderate Use/Low Morbidity. Black and women PWH were more likely to belong to the Moderate Use/High Cardiometabolic Burden class, while men who have sex with men were less likely to belong to the Heavy Use/Complex Behavioral Multimorbidity class. Classes differed in care outcomes: the Low Use/High Medical Morbidity class had the highest HIV care retention and viral suppression; the Heavy Use/Complex Behavioral Multimorbidity class had the highest alcohol treatment receipt; and the Moderate Use/High Cardiometabolic Burden class had the lowest psychotherapy rates.

**Conclusions:**

Syndemic patterns of alcohol use and multimorbidity among PWH were associated with disparities in HIV outcomes and alcohol treatment. Findings highlight missed opportunities for integrated alcohol and cardiometabolic care and support targeted interventions to improve engagement.

## Graphical abstract

Among 4574 adults with HIV in Kaiser Permanente Northern California, latent class analysis identified four syndemic classes combining alcohol use with co‐occurring conditions: Heavy Use/Complex Behavioral Multimorbidity, Moderate Use/High Cardiometabolic Burden, Low Use/High Medical Morbidity, and Moderate Use/Low Morbidity. Class membership varied by race/ethnicity, sex, and HIV risk group, and classes differed markedly in HIV care outcomes and alcohol treatment receipt. Findings reveal inequities in care engagement and highlight missed opportunities for integrated alcohol and cardiometabolic care among people with HIV.

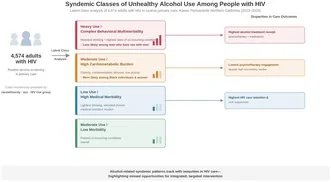

## 1 Introduction

Unhealthy alcohol use, defined by consumption that exceeds National Institute on Alcohol Abuse and Alcoholism (NIAAA) guidelines for daily and weekly consumption (> 3/4 drinks/day or > 7/14 drinks/week women and men 65+/men < 65; Donroe and Edelman 2022; National Institutes of Health Office of Aids Research 2020; Saitz 2005; Satre, Metz, et al. 2025), is common among PWH. Unhealthy alcohol use in PWH is associated with poorer antiretroviral therapy adherence, reduced retention in care and viral suppression (Satre, Sarovar, et al. 2025), increased HIV transmission risk behaviors (Satre et al. 2020), and higher mortality (Azar et al. 2010; Fuster et al. 2025; Ingle et al. 2025). Beyond its direct effects on HIV outcomes, unhealthy alcohol use frequently co‐occurs with other substance use, mental health disorders, sexually transmitted infections, cardiometabolic diseases (e.g., heart disease, obesity, diabetes), and chronic medical conditions (e.g., Castillo‐Carniglia et al. 2019; Gomez et al. 2023), contributing to high levels of multimorbidity among PWH. These co‐occurring conditions complicate clinical management and may undermine the effectiveness of HIV and behavioral health interventions delivered in routine care settings, such as brief alcohol interventions, antiretroviral therapy adherence support programs, and integrated behavioral health services (McKinnon et al. 2024).

Syndemics are defined as the co‐occurrence of two or more health conditions within a population, driven by social and structural inequities and interacting synergistically to amplify disease burden (Singer et al. 2006). Syndemic theory provides a useful framework for understanding how unhealthy alcohol use intersects with other behavioral, medical, and social conditions to worsen health outcomes among people with HIV (PWH; Mendenhall et al. 2022). Among PWH, alcohol use has been shown to co‐occur with smoking, depression (Sullivan et al. 2011), substance use disorders, sexually transmitted infections (Shuper et al. 2009), obesity (Obry‐Roguet et al. 2018), and cardiometabolic disease (Chichetto et al. 2021), all of which are associated with worse HIV outcomes such as higher or uncontrolled viral load (Chichetto et al. 2025). From a psychological perspective, these syndemics reflect intertwined behavioral and emotional processes shaped by stress, HIV stigma, and other barriers to care, rather than isolated risk factors (Pantalone et al. 2020).

Importantly, patterns of alcohol‐related multimorbidity may not be experienced equally across populations. Structural inequities related to race, ethnicity, sex, and HIV risk group shape exposure to chronic stressors, access to care, and vulnerability to alcohol‐related harms, and may influence how syndemic conditions manifest and impact health outcomes (Padilla et al. 2024). Prior research suggests that Black and Latine PWH experience disproportionate burdens of cardiometabolic disease and poorer HIV outcomes, and that alcohol‐related harms may be greater even at comparable levels of consumption (Aral et al. 2008; Naimi et al. 2025; Padilla et al. 2024). However, relatively little is known about whether distinct patterns of unhealthy alcohol use and co‐occurring conditions differ across sociodemographic groups, or how such differences relate to HIV care outcomes. Understanding these patterns may be highly valuable to aid in developing personalized interventions that could also reduce well‐known sociodemographic disparities.

However, despite growing recognition that unhealthy alcohol use commonly co‐occurs with multiple mental health and medical conditions among PWH, less is known about how these conditions cluster within individuals in clinically meaningful ways. Most prior studies rely on variable‐centered approaches that examine alcohol use, comorbidities, and HIV outcomes independently, which may obscure patterns of co‐occurring risk that shape treatment needs and response. Person‐centered analytic approaches offer a promising strategy to address these gaps. Latent class analysis (LCA) is a statistical method that identifies subgroups of individuals who share similar patterns of characteristics (Lanza 2016), allowing for the examination of complex, co‐occurring risk classes that align closely with syndemic frameworks (Hill et al. 2019). LCA has been increasingly applied in HIV and substance use research to characterize heterogeneity in risk behaviors and health conditions (Scheer et al. 2021), yet few studies have applied this approach to examine syndemics of alcohol use and multimorbidity among PWH within large, integrated health systems. Moreover, few studies have evaluated whether these syndemic classes are differentially distributed across demographic groups or associated with disparities in receipt of behavioral health services and HIV care outcomes.

Integrated health systems with systematic alcohol screening provide a unique opportunity to examine these questions using real‐world clinical data. Routine alcohol screening embedded in primary and HIV care allows for the identification of unhealthy drinking across the full spectrum of alcohol use, including those who report relatively moderate drinking who may nonetheless experience substantial medical or behavioral health burden (Justice et al. 2016; Palzes, Parthasarathy, et al. 2020; Sterling et al. 2020). When combined with electronic health record data on mental health diagnoses, substance use disorders, cardiometabolic risk factors, and HIV outcomes, these systems enable comprehensive assessment of syndemic risk patterns and their clinical implications at scale (Palzes et al. 2024).

The present study applies LCA to identify syndemic classes of alcohol use and multimorbidity among PWH receiving care in a large integrated health system. We aimed to: (1) identify and characterize distinct patterns of alcohol use, behavioral health conditions, and medical comorbidity; (2) examine whether race, ethnicity, sex, and HIV risk status were associated with membership in these syndemic classes; and (3) assess whether class membership is associated with HIV care outcomes and receipt of alcohol treatment. By integrating syndemic theory with a person‐centered analytic approach, this study seeks to clarify how alcohol use patterns and multimorbidity intersect to shape disparities in alcohol treatment as well as HIV care engagement and viral suppression among PWH receiving care in an integrated health system, with implications for more equitable and tailored intervention strategies in clinical practice.

## 2 Materials and Methods

### 2.1 Study Design and Setting

This study used electronic health record (EHR) data from Kaiser Permanente Northern California (KPNC), a large, integrated, nonprofit healthcare system providing comprehensive medical and behavioral health care to over 4.5 million members. The membership is diverse and reflects the insured population in the United States (Davis et al. 2023; Gordon 2020), with coverage provided through employer‐based plans, Medicare, Medicaid, and health insurance exchanges. KPNC has implemented routine, systemwide screening for unhealthy alcohol use in adult primary care settings (which includes HIV clinics), with results integrated into the EHR (Silverberg et al. 2020; Sterling et al. 2025). KPNC also maintains a comprehensive HIV care registry that captures demographic characteristics, clinical diagnoses, laboratory values, antiretroviral therapy use, and healthcare utilization. This registry is populated through electronic monitoring of inpatient, outpatient and laboratory testing databases (Silverberg et al. 2024). Data for the present analyses were drawn from the KPNC EHR and the KPNC HIV registry.

KPNC has a multidisciplinary, team‐based approach to HIV care emphasizing holistic, patient‐centered care to improve engagement and clinical outcomes (Horberg et al. 2020). Psychiatry and addiction medicine treatment in KPNC includes assessment, individual and group therapy, and medication management. Addiction medicine services further include day hospital, education, and relapse prevention groups. KPNC members can access psychiatry and addiction medicine services without referral, except members with Medicaid coverage, who must access addiction medicine services through county providers (Gordon 2020).

### 2.2 Study Sample

The analytic sample included adult PWH from the HIV registry aged 18 years or older who were active KPNC members and reported any alcohol use on routine primary care alcohol use screening between July 1, 2013 and December 31, 2020. July 2013 corresponds to the rollout of standardized alcohol screening across KPNC. The index date was defined as the first alcohol screening during this period in which the participant reported any alcohol use in the prior 3 months. Participants were required to have continuous KPNC membership for at least 1 year prior to the index date to assess latent class indicators to assess HIV and alcohol‐related outcomes. The final analytic sample consisted of 4574 PWH who met these criteria.

### 2.3 Measures

#### 2.3.1 Alcohol Use

Alcohol use was assessed via annual primary care screening by medical assistants using National Institute on Alcohol Abuse and Alcoholism (NIAAA) guidelines (National Institute on Alcohol Abuse and Alcoholism 2025). Questions include heavy drinking in the prior 90 days, usual quantity and usual frequency of alcohol use in standard drinks. Screening responses were used to derive measures of whether participants exceeded recommended daily limits, weekly limits, both limits, or neither, based on age‐ and sex‐specific NIAAA thresholds (Chi et al. 2022). Exceeding daily limits (heavy drinking) was defined as ≥ 5 drinks per day for men aged 18–65 years or ≥ 4 drinks per day for men aged ≥ 66 years and for women of any age. Exceeding weekly limits was defined as average weekly alcohol consumption ≥ 15 drinks per week for men age 18‐65 or ≥ 8 drinks per week for men age 66+ and for women of any age, calculated from reported drinking frequency and typical quantity. Using these indicators, a single categorical alcohol use variable was constructed with four mutually exclusive levels: (1) drinking within recommended NIAAA limits, (2) exceeds daily limits only, (3) exceeds weekly limits only, and (4) exceeds both daily and weekly limits.

#### 2.3.2 Latent Class Indicators

Behavioral health and medical comorbidity indicators were entered into latent class models (as described below). This included ICD‐9 and ICD‐10 diagnostic variables extracted from the EHR for the one‐year period prior to the index alcohol screening date. Behavioral health indicators included alcohol use disorder (AUD) diagnosis, substance use disorder (SUD) diagnosis (excluding alcohol and tobacco), and any diagnosed mental health condition, including attention‐deficit/hyperactivity disorder, anxiety disorder, obsessive compulsive disorder, post‐traumatic stress disorder, anorexia nervosa, bulimia nervosa, bipolar disorder, depression, other mood disorders, schizoaffective disorder, schizophrenia, other psychoses, and trauma‐ and stressor‐related disorders (Palzes, Parthasarathy, et al. 2020; Palzes, Weisner, et al. 2020). Current smoking status was assessed from self‐reported status from primary care screenings.

We created a dichotomized variable reflecting the presence of any of a set of chronic medical conditions selected from prior literature examining diagnoses commonly reported among individuals with alcohol use, including arthritis, asthma, atrial fibrillation, atherosclerosis, cerebrovascular disease, chronic kidney disease, chronic liver disease, chronic obstructive pulmonary disease, chronic pain, coronary disease, dementia, diabetes, epilepsy, gastroesophageal reflux, heart failure, hyperlipidemia, hypertension, migraine, osteoporosis, Parkinson's disease, and peptic ulcer (Ornstein et al. 2013; Sterling et al. 2020). These specific diagnoses were selected based on prior research documenting their association with alcohol use problems (Ornstein et al. 2013; Sterling et al. 2020); this indicator reflects conditions commonly co‐occurring with alcohol use in the literature. Cardiometabolic health indicators based on EHR data included obesity (body mass index ≥ 30 kg/m^2^) and insufficient physical activity (< 150 min of moderate physical activity per week). Physical activity is self‐reported via primary care screening in the prior year to alcohol screening. Sexually transmitted infection (STI) diagnoses included chlamydia, gonorrhea, and syphilis from one year prior to the index date through the index date. For each STI, we created a separate binary indicator, as well as an overall STI indicator. The overall STI flag was set to 1 if any of the three infections—chlamydia, gonorrhea, or syphilis—was present during the observation window; they were also included as an indicator of sexual health risk (Hojilla et al. 2020). All indicators were coded as binary variables reflecting the presence or absence of each during the one year pre‐index period, with an additional category indicating missing data where applicable.

#### 2.3.3 Predictors: Sociodemographic and HIV Risk Variables

Sociodemographic variables from the EHR included age at index date, sex (female, male), race and ethnicity (Asian, Black, Latine, White, Other, unknown), and socioeconomic status (SES). As prior research documents disparities in HIV‐related and behavioral health outcomes, separate contrasts were examined for Black versus non‐Black individuals and for Latine versus non‐Latine individuals. Sex was coded as 1 = female and 0 = male. HIV risk status was derived via structured chart review of electronic health records (EHR) by trained medical records analysts, who abstract documented indicators of HIV transmission risk (e.g., sexual activity patterns, sexual orientation, and injection drug use). Risk indicators are initially coded as separate binary variables (e.g., injection drug use; sexual activity consistent with men who have sex with men [MSM]). These indicators are then collapsed into a single, mutually exclusive categorical variable using a predefined hierarchical ranking algorithm applied within the health system. The final analytic variable reflects this mutually exclusive classification, with 1 = MSM and 0 = all other risk categories. Additional internal variables include a quantitative coding of risk category, a detailed categorical classification, and a collapsed categorical classification, of which the latter was used for the present analyses. As a proxy measure of SES, we used the neighborhood deprivation index (NDI) from geocoded census‐tract data from the 2019 American Community Survey and created a categorical variable based on quartiles of the samples' distribution, in which the first quartile reflected the least deprived neighborhood (higher SES) and the fourth quartile reflected the most deprived neighborhood (lower SES; Messer et al. 2006).

#### 2.3.4 Outcomes: HIV Care and Alcohol Treatment Receipt

Viral suppression was defined as HIV RNA < 200 copies/mL, measured between 3 months prior to and 12 months following the index date. HIV viral control was measured using data from the KPNC HIV registry. Retention in HIV care was defined as having at least two HIV‐related clinical encounters or laboratory measurements separated by at least 60 days within the follow‐up period (Satre, Sarovar, et al. 2025). We examined two AUD treatment types: behavioral intervention and medication. Receipt of was defined as having ≥ 1 encounter in the year post‐index for individual or group psychotherapy with a provider in psychiatry or addiction medicine, captured using CPT codes 90792, 90832–90840, 90845, and 90853. We did not include codes for evaluation without medical services (CPT 90791), or for family psychotherapy (CPT 90846, 90847). Medication use was defined as having ≥ 1 dispensed prescription in the year post‐index for a medication approved by FDA (acamprosate, disulfiram, naltrexone) or used off‐label (baclofen, gabapentin, nalmefene, topiramate) to treat AUD (Kranzler and Soyka 2018). Off‐label medications were only included if they were prescribed by a provider in psychiatry or addiction medicine, to avoid capturing prescriptions for other indications. For each calendar year, medications dispensed the previous year were counted if supply was sufficient to last until the current year (e.g., a 90‐day supply dispensed on 1 November of the previous year; Davy‐Mendez et al. 2021).

### 2.4 Statistical Analysis

Data analysis proceeded through the recommended classify‐analyze multistep process for identifying latent classes and their associations with outcomes (Dziak et al. 2016). First, latent class analysis (LCA) was used to select the optimal number of latent classes (i.e., subgroups) and then to identify and describe subgroups based on distinct patterns of alcohol use and multimorbidity. LCA was conducted on the full sample of PWH reporting any alcohol use. To select the optimal latent class model, the following criteria were considered: Akaike information criterion (AIC; Akaike 1974), Bayesian information criterion (BIC; Schwarz 1978), sample size adjusted BIC (a‐BIC; Sclove 1987), Vuong‐Lo–Mendell–Rubin Likelihood ratio test (VLMR; Dziak et al. 2016; Nylund et al. 2007), and entropy (Muthén 2004), as well as theoretical interpretability and model stability (Lanza, Tan, and Bray 2013). Lower AIC, BIC, and a‐BIC values indicated more optimal model fit, and higher entropy values indicated higher classification utility (Lanza, Bray, and Collins 2013). Significance of the VLMR likelihood ratio tests indicated whether the additional class in each successive model resulted in a significant improvement in model fit compared to the prior model with one fewer classes (Nylund et al. 2007). Model selection was guided by a combination of statistical fit indices, classification quality, and substantive interpretability. In cases where fit indices provided conflicting guidance, preference was given to more parsimonious solutions that demonstrated clear separation between classes, adequate class sizes, and clinical interpretability consistent with syndemic theory. Final model selection prioritized solutions in which classes reflected meaningful patterns of unhealthy alcohol use and multimorbidity with relevance for HIV care outcomes, rather than marginal improvements in fit achieved by adding additional parameters.

To interpret and describe the latent classes, two sets of parameters were examined: (1) latent class membership probabilities that represent class prevalences in the sample and (2) item response probabilities conditional on class membership. Latent class separation (i.e., distinction between classes as unique patterns; Collins and Lanza 2009) was determined by comparing item response probabilities between classes and in contrast to the overall prevalence and visual/manual comparison across latent patterns holistically. The patterns of item response probabilities were used to interpret and name the classes.

Second, we used the R3STEP command in MPlus to conduct multinominal logistic regressions testing age, race/ethnicity, sex, and HIV risk status predictors of class membership. We use the term “predictors” consistent with standard terminology in the latent class literature for LCA with covariates (Lanza, Tan, and Bray 2013), in which demographic and other auxiliary variables are entered as predictors of latent class membership after the class structure has been estimated from the indicator variables. Third, using modal class assignment based on posterior probabilities and measurement error‐based weighting (Bolck, Croon and Hagenaars or BCH approach; Bakk and Vermunt 2016; Bolck et al. 2004), we examined differences across four outcome variables: viral suppression, retention in HIV care, behavioral intervention for alcohol use and prescription for AUD treatment medication. The BCH approach estimates class‐specific outcome proportions and associated standard errors that account for classification uncertainty, and tests overall differences across latent classes using chi‐squared tests. When overall tests were statistically significant, we conducted pairwise comparisons between classes to identify specific group differences. Statistical significance was defined using a two‐sided alpha level of *p* < 0.05. Model estimation using maximum likelihood with robust standard errors was conducted in Mplus (Version 7.4, Muthén and Muthén 1989–2018). To reduce the likelihood of convergence on local maxima and to ensure model identification, we used 1000 random sets of starting values in the initial stage and retained the 500 best solutions for final stage optimization. Consistency of the best log‐likelihood values across random starts was used to confirm that the global maximum likelihood solution was identified.

Missing data on LCA indicators was handled through full information maximum likelihood and all person records providing data on at least one LCA indicator were used in latent class identification. For LCA with covariates and outcomes, complete case analysis was employed and records missing health outcome or covariate data were excluded from regression models.

## 3 Results

### 3.1 Characteristics of PWH Who Report Alcohol Use

The analytic sample included 4574 people with HIV who reported alcohol use, with a mean age of 46.4 years (SD = 12.4) (Table 1). The sample was predominantly male (94.3%), with females comprising 5.7%. Participants identified primarily as White (54.9%), followed by Latine (17.6%), Black (13.5%), and Asian (6.7%), with smaller proportions identifying as other or unknown race/ethnicity. Most individuals had commercial insurance (84.4%), and the majority were classified as men who have sex with men (77.6%), with smaller proportions reporting heterosexual exposure (9.9%) or injection drug use (4.8%). Clinically (Table 3, Overall prevalence), most participants reported alcohol use within NIAAA guidelines (77.0%), while 23.0% exceeded daily and/or weekly limits, including 16.0% who exceeded daily limits only, 4.0% who exceeded weekly limits only, and 3.0% who exceeded both. Current smoking was reported by 19.0% of the sample, 10.0% had a documented AUD diagnosis, and 12.0% had a non‐alcohol non‐tobacco SUD diagnosis. Chronic medical conditions were common, affecting 52.0% of participants, and over one quarter had a documented mental health diagnosis (26.0%). Nearly half of the sample reported insufficient physical activity (49.0%), 19.0% met criteria for obesity, and 22.0% had a recent sexually transmitted infection.

**TABLE 1:** Demographic characteristics of the analytic sample (*N* = 4574).

	M or N	SD or %
Age	46.37	12.44
Sex		
Men	4312	94.27
Women	262	5.73
Race/Ethnicity		
Black	619	13.53
Latine	805	17.60
White	2511	54.90
Asian	304	6.65
Other	146	3.19
Unknown	189	4.13
Insurance		
Commercial	3860	84.39
Medicare	576	12.59
Medicaid	95	2.08
Other	43	0.94
Risk status		
Heterosexual	454	9.93
Injection drug use	219	4.79
Men who have sex with men (MSM)	3548	77.57
Other	26	0.57
Unknown	327	7.15
Neighborhood Deprivation Index		
1 = not at all deprived	1294	28.29
2 = a little deprived	1160	25.36
3 = somewhat deprived	1110	24.27
4 = very deprived	989	21.62

*Note:* The analytic sample included people with HIV (PWH) who reported any alcohol use in the prior 3 months.

### 3.2 Co‐Occurring Risk Patterns in PWH Who Drink Alcohol

For latent class model selection, models with 1–6 classes were considered. As shown in Table 2, the AIC continued to decrease without minimization as additional classes were added, which is a common occurrence with LCA (e.g., Bray et al. 2014). The BIC and a‐BIC were minimized at the four‐class model. VLMR values indicated adding classes improved model fit through the 4‐class model (*p* < 0.001) but no significant improvements from the 4‐ to 5‐class model (*p* = 0.30) or from the 5–6 class model. Meaningful reductions in fit criteria slowed for models with four or more classes. Thus, we considered the 4‐class model optimal for interpretation and further analysis.

**TABLE 2:** Model fit information and criteria for latent profile models with 1 to 6 profiles.

No. of profiles	df	AIC	BIC	a‐BIC	Entropy	VLMR (*p*)
1	11	43982.83	44053.54	44018.59	1.00	—
2	23	43125.83	43273.68	43200.59	0.73	< 0.001
3	35	42975.71	43200.69	43089.48	0.48	< 0.001
**4**	**47**	**42923.51**	**43225.63**	**43076.28**	**0.68**	**< 0.001**
5	59	42889.43	43268.69	43081.21	0.70	0.23
6	71	42862.67	43319.07	43093.45	0.71	0.15

*Note:* Bold indicates the selected latent profile model. *N* = 4574.

Abbreviations: a‐BIC, sample size adjusted Bayesian information criterion; AIC, Akaike information criterion; BIC, Bayesian information criterion; df, degrees of freedom; VLMR, Vuong‐Lo–Mendell–Rubin likelihood ratio test.

Latent class prevalences and item response probabilities conditional on the four classes are presented in Table 3. Throughout, class labels using terms such as “high” or “low” for a given indicator (e.g., medical comorbidity) refer to a relatively high or low estimated probability of endorsing that indicator within the class, given the dichotomous nature of each indicator. Class labels using the terms “low,” “moderate,” and “heavy” alcohol use describe each class's alcohol use pattern relative to the other classes, based on the proportion of members in each of the four NIAAA‐derived alcohol use categories (see Measures); these labels do not correspond to NIAAA's own drinking‐level categories. Notably, drinking within recommended limits was the modal response even within the Heavy Use/Complex Behavioral Multimorbidity class (67%); as described below, this class is more strongly distinguished by the clustering of AUD, SUD, and other behavioral health diagnoses than by the absolute proportion of members exceeding alcohol limits. Prototypical members of the first class, named *Heavy Use/Complex Behavioral Multimorbidity* (15% prevalence; *n* = 676), had relatively greater proportion of exceeding recommended NIAAA alcohol use limits across all dimensions (daily, weekly, and both); high rates of smoking, mental health, AUD, SUD, and chronic medical diagnoses; along with average prevalence of STI. We use “complex behavioral multimorbidity” to refer to the co‐occurrence of multiple behavioral health conditions—smoking, mental health diagnoses, AUD, and SUD—consistent with the syndemic clustering described above, and to distinguish this pattern from the medical/cardiometabolic multimorbidity characterizing other classes. Prototypical members of the second class, named *Moderate Use/High Cardiometabolic Burden* (13%; *n* = 599), had a pattern uniquely characterized by relatively higher than average rates of insufficient physical activity (63%) and obesity (100%), and relatively lower than average prevalence of smoking, AUD, SUD, and mental health conditions, along with average prevalence on the other indicators. Prototypical members of the third class, named *Low Use/High Medical Morbidity* (23%; *n* = 988), had a pattern of characterized by relatively greater than average prevalence of drinking within recommended limits (87%), lower rates of smoking, AUD, SUD, obesity, STI diagnoses; but with 100% chronic medical condition diagnosis. Finally, prototypical members of the fourth class, named *Moderate Use/Low Morbidity* (51%; *n* = 2311), had moderate alcohol use (as indicated by 75% drinking within recommended limits), relatively lower than average prevalence of AUD, SUD, alcohol‐related medical conditions, mental health diagnoses, and low cardiometabolic risk—lower rates of insufficient physical activity and 0% obesity prevalence—along with average smoking and slightly higher than average STI prevalence.

**TABLE 3:** Parameter estimates for the four‐class model.

		1. Heavy use complex behavioral multimorbidity	2. Moderate use high cardiometabolic burden	3. Low use high medical morbidity	4. Moderate use low morbidity
		Latent class proportions			
		0.15	0.13	0.22	0.51
		(*n* = 676)	(*n* = 599)	(*n* = 988)	(*n* = 2311)
	Overall prevalence	Item response probabilities within each class			
Alcohol use level					
Within limits	0.77	0.67	0.78	0.88	0.75
Exceeds only daily limits	0.16	0.18	0.18	0.06	0.19
Exceeds only weekly limits	0.04	0.07	0.02	0.05	0.03
Exceeds both daily/weekly limits	0.03	0.08	0.01	0.01	0.03
Current smoking					
Yes	0.19	0.35	0.13	0.06	0.21
No	0.81	0.65	0.87	0.94	0.79
AUD					
Yes	0.10	0.55	0.01	0.03	0.02
No	0.90	0.45	0.99	0.97	0.98
SUD					
Yes	0.12	0.61	0.02	0.05	0.03
No	0.88	0.39	0.98	0.95	0.97
Chronic medical condition					
Yes	0.52	0.68	0.52	1.00	0.28
No	0.48	0.32	0.49	0.00	0.72
Mental health					
Yes	0.26	0.53	0.17	0.31	0.18
No	0.75	0.47	0.83	0.69	0.82
Insufficient physical activity					
Yes	0.49	0.61	0.63	0.49	0.41
No	0.51	0.39	0.37	0.51	0.59
Obesity					
Yes	0.19	0.21	1.00	0.13	0.00
No	0.81	0.79	0.00	0.87	1.00
Any STI					
Yes	0.22	0.22	0.19	0.15	0.26
No	0.78	0.78	0.81	0.85	0.75

*Note: N* = 4574.

Abbreviation: STI, sexually transmitted infection.

### 3.3 Associations Between Age, Race/Ethnicity, Sex, and HIV Risk Status and Class Membership

Table 4 shows the odds of latent class membership by demographic groups and HIV risk status, relative to the Moderate Use/Low Morbidity class. First, compared with all other racial/ethnic groups, Black individuals had nearly twice the odds of belonging to the Moderate Use/High Cardiometabolic Burden class rather than the Moderate Low Use Morbidity class (OR = 1.96; 95% CI [1.48, 2.56]). In addition, Black individuals had lower odds of belonging to the Low Use/High Medical Morbidity class relative to the Moderate Use/Low Morbidity class (OR = 0.41; 95% CI [0.24, 0.70]) and relative to all other racial/ethnic groups. Second, Latine individuals were significantly less likely to belong to the Heavy Use/Complex Behavioral Multimorbidity Class (OR = 0.70; 95% CI [0.51, 0.95]) and the Low Use/High Medical Morbidity class (OR = 0.19; 95% CI [0.10, 0.38]) compared with all other racial/ethnic groups. Next, sex emerged as a significant predictor of class membership such that women (relative to men) were significantly more likely to belong to the Moderate Use/High Cardiometabolic Burden class (OR = 2.23; 95% CI [1.36, 3.67]) and the Low Use/High Medical Morbidity class (OR = 2.30; 95% CI [1.23, 4.29]) as compared to the reference class. Finally, men who have sex with men (MSM) were significantly less likely to belong to the Heavy Use/Complex Behavioral Multimorbidity class (OR = 0.48; 95% CI [0.74, 0.64]) relative to all other HIV risk groups and relative to the reference class. Age was also entered as a predictor of class membership but did not significantly predict membership in any class relative to the reference class (all *p* > 0.05).

**TABLE 4:** Odds of latent class membership by demographics and risk status.

	Moderate use low morbidity (Reference)	Heavy use complex behavioral multimorbidity	Low use high medical morbidity	Moderate use high cardiometabolic burden
		OR (95% CI)	OR (95% CI)	OR (95% CI)
Age	—	1.06 (1.04, 1.07)	1.16 (1.14, 1.18)	1.03 (1.02, 1.04)
Women	—	1.23 (0.71, 2.15)	2.30 (1.23, 4.29)**	2.23 (1.36, 3.67)**
MSM	—	0.48 (0.74, 0.64)**	0.86 (0.61, 1.22)	0.81 (0.60, 1.09)
Black	—	1.30 (0.91, 1.74)	0.41 (0.24, 0.70)**	1.96 (1.48, 2.56)**
Latine	—	0.70 (0.51, 0.95)*	0.19 (0.10, 0.38)**	1.00 (0.76, 1.30)

*Note:* **p < 0.05. Women vs. Men; MSM vs. all other risk statuses; Black vs. non‐Black; Latine vs. non‐Latine. Bold values indicate statistically significant findings.

### 3.4 Classes Differentially Predict HIV and Alcohol Care Outcomes

Proportions and standard errors for each HIV and alcohol care outcome within each latent class are presented in Table 5. Overall, there were significant differences across classes in terms of viral suppression (*χ* ^*2*^ = 27.17, *p* < 0.001), retention in HIV care (*χ* ^*2*^ = 23.66, *p* < 0.001), and alcohol intervention receipt, both psychotherapy (*χ* ^*2*^ = 42.59, *p* < 0.001) and medication for AUD (*χ* ^*2*^ = 8.74, *p* < 0.05). Specifically, the Low Use/High Medical Morbidity class had the highest rate of viral suppression (98%), significantly higher compared to all other classes; along with significantly higher retention in HIV care relative to Moderate Use/Low Morbidity and Moderate Use/High Cardiometabolic Burden classes. In addition, the Heavy Use/Complex Behavioral Multimorbidity class had higher retention in HIV care compared to the Moderate Use/Low Morbidity class. Finally, in terms of alcohol intervention, the Heavy Use/Complex Behavioral Multimorbidity class had the highest proportion of receiving alcohol specialty care including both psychotherapy (19%) and medication for AUD (2%); in the context of overall low rates of medication for AUD, as is common in primary care patients (Harris et al. 2013); and both of these classes were significantly greater compared to all other groups. Finally, the Moderate Use/High Cardiometabolic Burden class was the least likely to receive psychotherapy (4%), with post hoc contrasts showing that they were significantly lower than the Moderate Use/Low Morbidity class (7%).

**TABLE 5:** Proportions and standard errors of HIV and alcohol care outcomes for each latent class, chi‐squared tests, and pairwise comparisons between classes.

	χ2	*p‐*value	Class 1	Class 2	Class 3	Class 4	Significant pairwise comparisons
			Heavy use complex behavioral multimorbidity	Moderate use high cardiometabolic burden	Low use high medical morbidity	Moderate use low morbidity	
Viral suppression	27.17	< 0.001	0.91 (0.01)	0.93 (0.01)	0.98 (0.01)	0.93 (0.01)	1, 2, 4 < 3
Retention in HIV care	23.66	< 0.001	0.88 (0.02)	0.85 (0.02)	0.93 (0.02)	0.83 (0.01)	2, 4 < 3; 4 < 1
Alcohol intervention							
Behavioral	42.59	< 0.001	0.19 (0.02)	0.04 (0.01)	0.07 (0.01)	0.07 (0.007)	2,3,4 < 1; 2 < 4
Pharmacologic	8.74	0.033	0.02 (0.01)	0.003 (0.00)	0.001 (0.00)	0.004 (0.002)	2,3,4 < 1

*Note:* Behavioral intervention includes individual and group therapy visits in specialty addiction care and/or psychiatry.

## 4 Discussion

The present study identified four distinct classes of unhealthy alcohol use and multimorbidity among PWH receiving care in a large, integrated health system and demonstrated that these classes were differentially distributed by race/ethnicity, sex, and HIV risk status, with meaningful implications for HIV and alcohol‐related care outcomes. By applying a person‐centered analytic approach grounded in syndemic theory—that is, an approach that groups individuals based on shared patterns of co‐occurring behavioral, mental health, and medical conditions rather than examining each condition in isolation—findings move beyond single risk‐factor models to clarify how patterns of behavioral, mental health, and medical conditions co‐occur within individuals and potentially reinforce disparities in care engagement and viral suppression. By identifying specific multimorbidity profiles associated with differential risk of suboptimal engagement in care and viral non‐suppression, this work provides actionable evidence to guide more tailored screening, referral, and integrated treatment strategies for PWH with unhealthy alcohol use.

### 4.1 Syndemic Classes of Alcohol Use and Multimorbidity

The four classes reflected heterogeneous pathways through which alcohol use intersects with broader health burden among PWH. For example, the Heavy Use/Complex Behavioral Multimorbidity class represented a classic syndemic pattern, characterized relatively (though not majority) elevated rates of exceeding alcohol limits, high rates of smoking, mental health disorders, alcohol and other substance use disorders, and chronic medical conditions. Indeed, this class was distinguished less by the proportion of members drinking heavily in absolute terms—most members reported drinking within recommended limits—than by the marked concentration of AUD, SUD, and other behavioral health diagnoses relative to the other three classes. This constellation is consistent with prior work demonstrating strong classing of substance use and mental health conditions among PWH (Hwang et al. 2025), likely driven by shared biopsychosocial mechanisms such as chronic stress exposure, emotion dysregulation, and stigma‐related coping. Importantly, this class also showed relatively strong engagement in HIV and alcohol‐related care, suggesting that high behavioral health burden may increase clinical visibility and trigger referrals within integrated systems.

In contrast, the Moderate Use/High Cardiometabolic Burden class was defined not by severe alcohol use or mental health conditions, but by obesity, insufficient physical activity, and cardiometabolic risk in the context of relatively moderate alcohol use and comparatively low rates of diagnosed AUD, SUD, or mental health conditions. This class highlights a distinct and often underrecognized syndemic pathway in which alcohol use may exacerbate cardiometabolic risk without meeting traditional AUD severity thresholds (Justice et al. 2016). The Low Use/High Medical Morbidity class further underscores this point: despite drinking largely within recommended limits, individuals in this group had substantial medical burden and the highest rates of viral suppression and care retention, suggesting frequent healthcare contact for chronic disease management. Interestingly, this group mirrors the “sick quitters” phenomenon (Shaper et al. 1988), which refers to the observation that individuals with low or no current alcohol use may include a disproportionate number of people who reduced or stopped drinking because of declining health, rather than because low alcohol use directly causes better health. This reverse‐causation pattern can make abstinent or low‐use groups appear to have elevated illness burden in cross‐sectional data, independent of any direct effect of alcohol use itself (Shaper et al. 1988). In the present study, this may help explain why the Low Use/High Medical Morbidity class (despite drinking largely within recommended limits) showed substantial chronic disease burden alongside the highest rates of viral suppression and care retention, consistent with a pattern of reduced alcohol use tied to underlying health status and frequent healthcare contact rather than to health‐protective abstention itself. Finally, the Moderate Use/Low Morbidity class represented a comparatively healthier class, comprising over half the sample, with lower overall multimorbidity and intermediate outcomes. Notably, even within the comparatively lower risk Moderate Use/Low Morbidity class, a small proportion of individuals received behavioral intervention for alcohol use (7%). This likely reflects the clinical recognition that PWH face elevated vulnerability to alcohol‐related harm, including impaired antiretroviral therapy adherence, accelerated liver disease, and increased mortality risk, even at levels of use that would not meet conventional risk thresholds in the general population (Satre, Metz, et al. 2025). As such, offering alcohol treatment to PWH somewhat independent of use severity may represent an appropriately cautious clinical approach for this population, rather than an inconsistency in treatment targeting. Together, findings emphasize that alcohol‐related risk among PWH is not monolithic. Syndemic burden can arise through behavioral and mental health pathways, medical cardiometabolic pathways, or combinations of both, each with different implications for detection, treatment, and outcomes.

### 4.2 Disparities in Syndemic Class Membership

A central contribution of this study is the demonstration that syndemic classes were differentially distributed by race, sex, and HIV risk status in ways that may help explain persistent disparities in HIV outcomes even among those with access to health care. Black PWH and women were disproportionately represented in the Moderate Use/High Cardiometabolic Burden class. This finding aligns with broader evidence that Black individuals with HIV and women with HIV experience elevated cardiometabolic risk and multimorbidity (Batterham et al. 2024). Structural factors, including chronic stress, racism, gendered caregiving roles, and inequities in access to preventive health resources, likely contribute to this pattern.

Importantly, Black individuals were less likely to belong to the Low Use/High Medical Morbidity class, which had the most favorable HIV outcomes, suggesting that similar levels of medical burden may translate into different care trajectories across racial groups. This finding suggests that Black individuals may be less likely to fall into the subgroup combining lower alcohol risk with high engagement in medical care, a pattern associated with better HIV‐related outcomes. Latine individuals showed lower odds of membership in both the Heavy Use/Complex Behavioral Multimorbidity and Low Use/High Medical Morbidity classes relative to the Moderate Use/Low Morbidity class, indicating greater likelihood of being in the Moderate Use/Low Morbidity group. Notably, this group did not demonstrate the most favorable retention in HIV care, suggesting that lower documented morbidity may not necessarily reflect lower underlying need. Individuals in this class may appear healthier in EHR data, yet potentially have fewer opportunities for behavioral health conditions to be identified due to lower healthcare utilization or shorter duration of engagement in care. This pattern may reflect heterogeneity within Latine populations as well as potential differences in diagnosis, documentation, or treatment engagement within clinical settings.

Women were also more likely to belong to both the Moderate Use/High Cardiometabolic Burden and Low Use/High Medical Morbidity classes, underscoring that women with HIV may experience syndemic patterns that are more medically than behaviorally oriented. Findings are consistent with evidence that women with HIV are underdiagnosed for AUD, despite experiencing disproportionate alcohol‐related harm and cardiometabolic disease (Duko et al. 2019). MSM were associated with lower odds of Heavy Use/Complex Behavioral Multimorbidity in this sample, which may reflect higher engagement in preventive and behavioral health services among MSM within integrated HIV care settings, or the well‐known psychosocial challenges of women living with HIV and those who acquire HIV via injection drug use (Storholm et al. 2016).

### 4.3 Implications for HIV and Alcohol‐Related Care

Syndemic class membership was meaningfully associated with HIV care engagement, viral suppression, and alcohol treatment receipt, consistent with prior research demonstrating that alcohol use operates within a broader constellation of behavioral and medical comorbidities to shape HIV outcomes (Chichetto et al. 2025; Satre, Sarovar, et al. 2025). The Low Use/High Medical Morbidity class exhibited the highest rates of viral suppression and retention in HIV care, likely reflecting frequent and sustained contact with the healthcare system for management of chronic conditions. This pattern aligns with prior HIV care continuum studies showing that engagement in non‐HIV medical care can facilitate retention in HIV care and viral suppression, even among individuals with substantial comorbidity burden (Colasanti et al. 2016).

In contrast, the Moderate Use/High Cardiometabolic Burden class demonstrated poorer HIV care engagement despite elevated medical risk. Prior work has shown that unhealthy alcohol use, smoking, and cardiometabolic conditions are independently associated with reduced retention in care and viral suppression, particularly when these risks co‐occur (Satre, Sarovar, et al. 2025). Our findings extend this literature by demonstrating that even moderate levels of alcohol use, when embedded within a cardiometabolic risk class, may signal vulnerability for disengagement from HIV care, suggesting that traditional alcohol severity thresholds may inadequately capture risk for poor HIV outcomes. Alternatively, lower retention in this group may reflect the accumulation of structural and clinical vulnerabilities disproportionately borne by individuals in this class, including higher representation of women and racial/ethnic minoritized groups who face well‐documented barriers to consistent engagement in HIV care. Prior work by Justice and colleagues (Justice et al. 2016) suggests that among individuals with greater medical complexity, including PWH, alcohol‐related risk may occur at lower consumption thresholds than those typically used in general population guidelines. In this context, even moderate alcohol use may interact with co‐occurring cardiometabolic burden and structural barriers to care to compound risk for suboptimal HIV outcomes.

Patterns of alcohol treatment receipt varied substantially across syndemic classes, highlighting how pathways into alcohol care are shaped by comorbidity context rather than alcohol use alone. The Heavy Use/Complex Behavioral Multimorbidity class exhibited the highest rates of both behavioral and pharmacologic alcohol treatment, likely reflecting alignment with clinical pathways that prioritize treatment initiation among individuals with diagnosed AUD and co‐occurring mental health conditions. Consistent with prior studies in PWH, treatment initiation appears to be strongly patterned by symptom severity, mental health comorbidity, and patient characteristics, rather than alcohol use in isolation (Davy‐Mendez et al. 2021; Metz et al. 2025).

In contrast, individuals in the Moderate Use/High Cardiometabolic Burden class were the least likely to receive psychotherapy for alcohol, despite elevated medical risk and overrepresentation of Black individuals and women. This pattern mirrors findings from syndemic‐focused studies showing that co‐occurring conditions such as alcohol use, smoking, depression, and cardiometabolic risk may contribute to adverse outcomes without reliably triggering alcohol‐related intervention (Chichetto et al. 2021; Silverberg et al. 2024). Consistent with prior work demonstrating disparities in receipt of brief interventions among women and lower initiation of addiction treatment among Black patients (Lu et al. 2021; McCrady et al. 2020; Saloner and le Cook 2013), individuals in this class may face structural and clinical barriers that reduce the likelihood that alcohol use is addressed in care. In addition, cardiometabolic conditions such as obesity and hypertension may not be consistently recognized by patients or providers as indicators of elevated alcohol‐related risk when alcohol use falls within moderate ranges. Together, these findings underscore a critical gap in care: individuals whose alcohol use does not exceed guideline thresholds or diagnostic criteria but which may nonetheless contribute to clinically meaningful risk when occurring alongside other syndemic vulnerabilities. Collectively, these results highlight the need for more holistic, risk‐informed approaches to screening and intervention that account for the broader constellation of medical, behavioral, and structural factors shaping both HIV outcomes and access to alcohol‐related care (Womack and Justice 2020).

### 4.4 Clinical and Public Health Implications

These findings have several implications for clinical practice and health equity. First, routine alcohol screening should be interpreted within a broader syndemic context that includes cardiometabolic risk, physical activity, and obesity, particularly for women and Black PWH. Second, integrated HIV care models may benefit from expanding alcohol‐related interventions beyond AUD‐focused frameworks to include brief behavioral interventions and preventive counseling delivered across multiple points of clinical contact, not solely within primary care or specialty addiction settings. Embedding alcohol screening, brief intervention, and referral pathways within cardiometabolic care contexts, such as diabetes, hypertension, obesity, or cardiovascular disease clinics where many PWH with moderate drinking and high medical risk are routinely seen, may improve identification and address alcohol‐related barriers to chronic disease management and HIV outcomes (Silverberg et al. 2024; Sterling et al. 2026). Further, results may suggest that brief intervention may not be sufficient for people who have these comorbidities. For example, Satre et al. (2019) found that three session MI was better than brief intervention for people with comorbid drug use. Alternatively, SBIRT implementation may be lacking for individuals with cardiometabolic risk factors, who may be more likely to receive care at addiction medicine and recovery services. Third, culturally responsive approaches that address the needs of women with HIV and other marginalized groups are needed to address cardiometabolic syndemics that disproportionately affect these populations and may contribute to persistent disparities in HIV clinical outcomes.

From a psychological perspective, these results underscore the importance of addressing upstream stressors and behavioral pathways that link alcohol use, physical inactivity, and chronic disease. Multilevel interventions that integrate community‐based prevention, health system supports, and coordinated behavioral health and medical care may be particularly relevant for individuals in the Moderate Use/High Cardiometabolic Burden class and could help reduce inequities in HIV care engagement.

### 4.5 Strengths, Limitations, and Future Directions

Findings should also be considered in light of several limitations. The insured nature of the sample may limit generalizability to uninsured or underinsured populations with HIV. While alcohol use was measured using validated screening tools, some comorbid conditions relied on provider‐assigned diagnoses and may underestimate conditions that are underdiagnosed or stigmatized. Relatedly, alcohol use level and AUD/SUD diagnosis reflect different assessment windows and are not interchangeable indicators of severity: alcohol use level was derived from self‐reported screening referencing the prior 90 days, capturing a point‐in‐time consumption snapshot, whereas AUD and SUD diagnoses were drawn from ICD‐9/10 codes documented over the one‐year period prior to the index screening date and may reflect a longer‐standing or more severe drinking history, consequences, or prior treatment episodes not reflected in current reported consumption. A diagnosis may also persist in the EHR even after reported use has declined, for example following treatment or natural recovery. The cross‐sectional measurement of latent class indicators precludes causal inference regarding the development or temporal ordering of syndemic conditions. In addition, the relatively small proportion of women limited more nuanced examination of gender diversity and intersectional identities. Future research should examine longitudinal transitions between syndemic classes, incorporate social determinants of health and measures of stress and stigma, and test whether class‐informed, multilevel interventions improve HIV and alcohol‐related outcomes. Further, the current analysis characterized medical and mental health comorbidity using composite dichotomous indicators, reflecting the presence versus absence of any condition within each domain. This approach was chosen to preserve model parsimony and estimation stability given the large number of individual diagnoses represented in each category (17 mental health diagnoses; 26 medical diagnoses), many of which individually occur at low prevalence in this sample. As a result, the current classes reflect broad comorbidity burden rather than distinctions between specific diagnostic subtypes (e.g., depression vs. anxiety, or cardiovascular ves. metabolic conditions). Future work with larger samples, or samples enriched for specific diagnostic subgroups, could examine whether more fine‐grained diagnostic distinctions reveal additional heterogeneity in risk profiles not captured by the composite indicators used here. In addition, socioeconomic status was measured using a neighborhood deprivation index derived from 2019 American Community Survey data, applied uniformly across the 2013–2020 study period; while neighborhood‐level socioeconomic characteristics tend to be relatively stable over multiyear periods, some misclassification of SES for participants screened earlier in the study period cannot be ruled out. Finally, data collection spanned July 2013 through December 2020, and the final year overlapped with the onset of the COVID‐19 pandemic, during which disruptions to routine primary care visits, alcohol screening, and health care utilization more broadly may have affected both the identification of the analytic sample and the measurement of care outcomes for participants screened during this period.

This study also has several notable strengths, including a very large sample of people with HIV drawn from an integrated health system with comprehensive linkage across alcohol screening, HIV clinical outcomes, comorbid conditions, and health care utilization. Alcohol use was assessed using routine, systematic screening with validated measures, strengthening confidence in alcohol‐related outcomes and minimizing selection bias common to non‐universal screening approaches. The integrated care setting also enabled examination of syndemic patterns in relation to clinically meaningful outcomes such as retention in care and viral suppression.

## 5 Conclusion

The present study demonstrates that unhealthy alcohol use among PWH is associated with distinct syndemic classes that are patterned by race, sex, and HIV risk status and differentially associated with HIV and alcohol care outcomes. Black PWH and women were disproportionately represented in a cardiometabolically burdened class that was least likely to receive behavioral alcohol intervention, highlighting missed opportunities for equitable, integrated care. Taken together, the identified syndemic classes highlight how co‐occurring unhealthy alcohol use and multimorbidity may undermine retention in HIV care and sustained viral suppression, underscoring the need for integrated approaches that extend beyond antiretroviral therapy alone. Addressing alcohol use within a syndemic framework that accounts for multimorbidity and structural inequities may be essential for reducing disparities and improving long‐term outcomes for PWH.

## Funding

Dr. Van Doren was supported by the National Institute on Drug Abuse (T32DA007250) and the National Institute on Alcohol Abuse and Alcoholism (K23AA033108). Dr. Satre was supported by the National Institute on Alcohol Abuse and Alcoholism (K24AA025703).

## Conflicts of Interest

The authors declare no conflicts of interest.

## Data Availability

The data that support the findings of this study are available on request from the corresponding author. The data are not publicly available due to privacy or ethical restrictions.
